# Body mass index and two-year change of in vivo Alzheimer’s disease pathologies in cognitively normal older adults

**DOI:** 10.1186/s13195-023-01259-w

**Published:** 2023-06-13

**Authors:** Seunghoon Lee, Min Soo Byun, Dahyun Yi, Min Jung Kim, Joon Hyung Jung, Nayeong Kong, Gijung Jung, Hyejin Ahn, Jun-Young Lee, Koung Mi Kang, Chul-Ho Sohn, Yun-Sang Lee, Yu Kyeong Kim, Dong Young Lee, for the KBASE Research Group

**Affiliations:** 1 Department of Psychiatry, Myongji Hospital, Hanyang University College of Medicine, Goyang, 10475 Republic of Korea; 2 Department of Neuropsychiatry, Seoul National University Hospital, Seoul, 03080 Republic of Korea; 3 Department of Psychiatry, Seoul National University College of Medicine, Seoul, 03080 Republic of Korea; 4 Institute of Human Behavioral Medicine, Medical Research Center, Seoul National University, 101 Daehak-Ro, Jongno-Gu, Seoul, 03080 Republic of Korea; 5 Department of Neuropsychiatry, Nowon Eulji University Hospital, Seoul, 01830 Republic of Korea; 6 Department of Neuropsychiatry, SMG-SNU Boramae Medical Center, Seoul, 07061 Republic of Korea; 7 Department of Radiology, Seoul National University Hospital, Seoul, 03080 Republic of Korea; 8 Department of Nuclear Medicine, Seoul National University College of Medicine, Seoul, 03080 Republic of Korea; 9 Department of Nuclear Medicine, SMG-SNU Boramae Medical Center, Seoul, 07061 Republic of Korea

**Keywords:** Body mass index, Alzheimer disease, Beta-amyloid, Tau, Longitudinal changes

## Abstract

**Background:**

Low body mass index (BMI) or underweight status in late life is associated with an increased risk of dementia or Alzheimer’s disease (AD). However, the relationship between late-life BMI and prospective longitudinal changes of in-vivo AD pathology has not been investigated.

**Methods:**

This prospective longitudinal study was conducted as part of the Korean Brain Aging Study for Early Diagnosis and Prediction of Alzheimer’s Disease (KBASE). A total of 194 cognitive normal older adults were included in the analysis. BMI at baseline was measured, and two-year changes in brain Aβ and tau deposition on PET imaging were used as the main outcomes. Linear mixed-effects (LME) models were used to examine the relationships between late-life BMI and longitudinal change in AD neuropathological biomarkers.

**Results:**

A lower BMI at baseline was significantly associated with a greater increase in tau deposition in AD-signature region over 2 years (β, -0.018; 95% CI, -0.028 to -0.004; *p* = .008), In contrast, BMI was not related to two-year changes in global Aβ deposition (β, 0.0002; 95% CI, -0.003 to 0.002, *p* = .671). An additional exploratory analysis for each sex showed lower baseline BMI was associated with greater increases in tau deposition in males (β, -0.027; 95% CI, -0.046 to -0.009; *p* = 0.007), but not in females.

**Discussion:**

The findings suggest that lower BMI in late-life may predict or contribute to the progression of tau pathology over the subsequent years in cognitively unimpaired older adults.

## Introduction

A large amount of evidence indicates that body mass index (BMI) is related to the risk of Alzheimer’s disease (AD) dementia [1–3]. Several studies have shown that being overweight or obese in midlife increases the risk of AD dementia or cerebral beta-amyloid (Aβ) deposition [4–6]. However, multiple studies have also reported that low BMI or being underweight in late life was associated with an increased risk of dementia [1, 3, 7] and that higher BMI in late life was a protective factor for AD dementia [3, 8].

Several amyloid positron emission topography (PET) studies with cross-sectional design demonstrated that lower BMI in late life was associated with increased brain Aβ burden in cognitive normal(CN) elderly individuals [9–12]. Other cross-sectional studies also reported a correlation between lower late-life BMI and increased CSF total tau or phosphorylated-tau [9, 13, 14]. A study has reported that there is a correlation between frailty and brain atrophy as measured by MR imaging, with greater frailty being associated with greater brain atrophy in community dwelling older adults [15]. All these findings are consistent with the association between low BMI in late life and a higher risk of AD dementia. In regard of longitudinal approach, some prospective studies have reported that brain Aβ is associated with future decreased of BMI, suggesting that weight loss, as well as cognitive decline, may be a clinical manifestation of AD process [16, 17]. However, the relationship between late-life BMI and prospective longitudinal changes of in-vivo AD pathology has not yet been investigated. Understanding such relationship of current BMI and future prospective changes of AD pathological biomarkers in cognitively unimpaired older adults could make it clearer whether lower BMI can predict or contribute to the progression of AD pathology and subsequently to AD dementia risk.

In this context, we tested the hypothesis that a lower late-life BMI is related to a greater prospective increase in in-vivo AD pathology, including Aβ and tau deposition, in cognitively healthy individuals. Additionally, as several previous studies showed prominent sex-related differences for the relationship between BMI and AD dementia risk [18, 19] and brain Aβ deposition [11, 20], we explored the same relationship for each sex separately.

## Methods

### Participants

This study was performed as part of the Korean Brain Aging Study for Early Diagnosis and Prediction of Alzheimer’s Disease (KBASE), an ongoing prospective cohort study conducted from 2014 [21]. As of 2018, 297 CN adults between 55 and 90 years old were recruited and received a baseline evaluation, including a comprehensive clinical assessment and BMI measurement. Among them, 194 participants who had completed both baseline and two-year follow-up neuroimaging scans for brain Aβ deposition were included in the current study. The inclusion criteria were as follows: (a) age 55–90 years, (b) Clinical Dementia Rating score of 0, and (c) no diagnosis of mild cognitive impairment or dementia. The exclusion criteria were as follows: (a) any serious medical, psychiatric, or neurological disorder that could affect mental function; (b) any severe communication problem that would render clinical examination or brain scanning difficult; (c) contraindications to magnetic resonance imaging (MRI), such as a pacemaker or claustrophobia; (d) absence of a reliable informant; (e) illiteracy defined as a lack of the ability to read; and (f) participation in another clinical trial or treatment with an investigational product. Research clinicians determined the presence of any exclusion criteria by referring to the results of laboratory examinations and MRI scans. They also evaluated the clinical data collected by trained nurses during systematic interviews of participants and their reliable informants during the screening period. More detailed information on the recruitment of the KBASE cohort has been presented in a previous report [21]. The study was approved by the Institutional Review Board of the Seoul National University Hospital and SNU-SMG Boramae Medical Center, South Korea. All participants provided written informed consent.

### Clinical assessment

The participants underwent comprehensive baseline clinical assessments based on the KBASE protocol [21] by trained psychiatrists. The assessments incorporated the Korean version of the Consortium to Establish a Registry for Alzheimer`s Disease Assessment (CERAD-K) [22, 23]. The presence of vascular risk factors (VRFs), including diabetes, hypertension, dyslipidemia, coronary heart disease, transient ischemic attack, and stroke, was assessed from data collected during systematic interviews by trained nurses with participants and their informants. Based on the number of VRFs, the vascular risk score (VRS) was calculated [24] and treated as a continuous variable for analyses. The Geriatric Depression Scale (GDS) [25] was used to measure the severity of depressive symptoms. Smoking status (never/former/smoker), alcohol intake status (never/former/drinker), and lifetime physical activity were evaluated through interviews with nurses. The Lifetime Total Physical Activity Questionnaire [26] was used to assess lifetime physical activity. A metabolic equivalent (MET) value was assigned to the intensity of activity based on the compendium of physical activities [27].

### BMI measurement

BMI was calculated as weight in kilograms divided by the square of the height in meters. It was measured at the baseline visit. Trained research nurses measured the participants’ height and body weight using standard anthropometric methods.

### Measurement of Aβ biomarker

All participants underwent [^11^C] Pittsburgh compound B (PiB)-positron emission tomography (PET) scans using a 3.0 T Biograph mMR (PET-MR) scanner (Siemens, Washington DC, USA). These scans were conducted according to the manufacturer’s protocols at baseline and two-year follow-up visit. We described the details of PiB-PET image acquisition and preprocessing previously [28]. The automatic anatomic labeling algorithm and the region combination method [29] were used to determine regions of interest (ROIs) and to characterize PiB retention in the frontal, lateral parietal, posterior cingulate-precuneus, and lateral temporal regions. A global cortical ROI (consisting of the four smaller ROIs) was also defined. The global Aβ retention value, the standardized uptake value ratio (SUVR) for the global cortical ROI, was calculated by dividing the mean values for all voxels of the global cortical ROI by a mean reference region. For the analysis of baseline data, the inferior cerebellar gray matter in the spatially unbiased infratentorial template for the cerebellum (SUIT) atlas [30] was used as the reference region. A participant was classified as Aβ positive if the SUVR was > 1.21 [31]. For longitudinal analysis, the reference region included the inferior cerebellar grey matter, cerebellar white matter (thresholded at 50%), pons, and cerebrum white matter (thresholded at 95% and eroded by three voxels) [32, 33].

### Measurement of cerebral tau deposition

A subset of subjects (*n* = 45) underwent two [^18^F] AV-1451 PET scans using a Biograph True Point 40 PET/CT platform (Siemens, USA) per the manufacturer’s guidelines at a two-year time interval. While the first PiB-PET imaging was performed during the baseline visit, the first AV-1451 PET imaging was performed at an average of 2.55 (standard deviation = 0.26) years after that visit. The details of AV-1451 PET imaging acquisition and preprocessing have been described previously [28]. We quantified the AV-1451 SUVR of a priori ROI of the “AD-signature region” of tau accumulation to estimate cerebral tau deposition. This was a size-weighted average of the partial volume-corrected uptake by the entorhinal, amygdala, parahippocampal, fusiform, inferior temporal, and middle temporal ROIs [34, 35]. It was done using the cerebral hemispheric white matter ROI from FreeSurfer in the partial volume code [36] as a reference region. The literature recommends using cerebral white matter as the reference region for intensity normalization in longitudinal AV-1451 PET data analysis [37].

### Statistical analyses

We tested linear mixed-effects (LME) models with random intercepts to examine the relationships between late-life BMI and longitudinal change in AD neuropathological biomarkers. All models included Aβ or tau deposition values as dependent variables on the first and second PET scan. Model 1 included baseline BMI, age, sex, APOE4, baseline Aβ or Tau and their interactions with time. In Model 2, we additionally controlled for VRS and its interaction with time to adjust for the confounding effects of vascular risk factors, considering the well perceived role of vascular risk factors in AD development [38, 39]. A random intercept was included for each subject, and time was calculated as the number of years from baseline. For exploratory purposes, the LME model including baseline BMI, age, APOE4, baseline Aβ or tau and their interactions with time was analyzed for each sex. Statistical analyses were performed using R version 4.0, and jamovi version 2.2.1 (The jamovi project, www.jamovi.org). In all analyses, *p* < 0.05 was considered as statistical significance.

### Availability of data and materials

The datasets generated and analyzed during the present study are not publicly available, owing to ethics considerations and privacy restrictions. Data might be obtained from the corresponding author after approval by the Institutional Review Board of the Seoul National University Hospital, South Korea.

## Results

### Participant characteristics

The demographic and clinical characteristics of all subjects are presented in Table 1.

**Table 1:** Participant characteristics

Variable	Total	Tau PET
No. of individuals	194	45
Age at baseline, year (mean ± SD)	68.4 ± 8.1	70.3 ± 7.3
Female, No. (%)	102 (53)	25 (55.6)
Education, year, median (IQR)	12 (7)	12(4)
APOE ε4 carriers, No. (%)	35 (18.0)	8 (17.8)
Baseline BMI, kg/m^2^ (mean ± SD)	24.20 ± 3.01	24.5 ± 2.55
Vascular risk factor, No. (%)		
Diabetes mellitus	35 (18.0)	10 (22.2)
Hypertension	87 (44.8)	21 (46.7)
Hyperlipidemia	69 (35.6)	15 (33.3)
Coronary heart disease	11 (5.7)	3 (6.7)
Stroke	0	0
TIA	1 (0.5)	1 (2.2)
VRS, median (IQR)	1 (0–2)	1 (0–2)
Alcohol use, No. (%)		
Never	98 (50.5)	25(55.6)
Former	23 (11.9)	7 (15.6)
Drinker	73 (37.6)	13 (28.9)
Smoking status, No. (%)		
Never	125 (64.4)	30 (66.7)
Former	57 (29.4)	13 (28.9)
Drinker	12 (6.2)	2 (4.4)
Lifetime physical activity, MET, median (IQR)	68.7 (57.2)	64.5 (42.1)
Cerebral Aβ deposition, SUVR		
Baseline global Aβ retention, median (IQR)	1.12 (0.11)	1.13 (0.11)
Baseline Aβ positive (> 1.20), No, (%)	43 (22)	13 (28.9)
Global Tau deposition, SUVR		
Baseline Tau retention, median (IQR)	1.02 (0.14)	1.00 (0.16)

*Abbreviations*: *Aβ* β-amyloid protein, *IQR* Interquartile range, *MET* metabolic equivalent, *SD* standard deviation, *SUVR* standardized uptake value ratio, *VRS* vascular risk score

### Association of BMI at baseline with cerebral Aβ and tau deposition change over two years

**Fig. 1:**
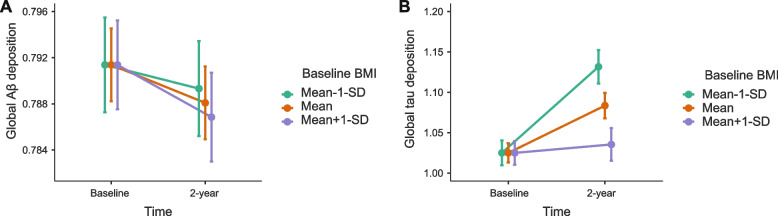
Changes of Global Amyloid and tau deposition over 2 years according to the baseline BMI strata. Estimates are from a linear mixed model predicting change in Aβ deposition (**A**) and in tau deposition (**B**). Controlling for age, sex, APOE4, baseline tau or Aβ and their interactions with time. Error bars represent standard error

Baseline BMI was not significantly associated with global Aβ deposition change during the two-year follow-up period for models 1 and 2. In contrast, a lower baseline BMI was significantly associated with a greater increase in tau deposition in the AD-signature region over two years (Table 2). When we conducted the same analyses including three BMI strata (below -1 SD, median BMI, above 1SD) instead of BMI as a continuous variable for the purpose of demonstration, the results were similar (Fig. 1 and Table 3). We also performed sensitivity analyses, including the GDS score, smoking status, alcohol intake status, and lifetime physical activity as additional covariates, which showed similar findings (Table 4).

**Table 2:** Association of the baseline BMI with neuroimaging biomarker changes for 2-year

	**Estimate**	**95% CI**	***t* value**	***p* value**
**Dependent variable: Aβ deposition**				
Model 1^a^				
Baseline BMI x time	0.000	-0.003 to 0.002	-0.359	.720
Model 2^b^				
Baseline BMI x time	0.000	-0.003 to 0.002	-0.426	.671
**Dependent variable: Tau deposition**				
Model 1^a^				
Baseline BMI x time	-0.018	-0.030 to -0.006	-3.027	.003
Model 2^b^				
Baseline BMI x time	-0.016	-0.028 to -0.004	-2.703	.008

*Abbreviations*: *Aβ* β-amyloid protein, *BMI* body mass index, *CI* confidence interval

^a^Adjusted for age, sex, APOE4, baseline Aβ or Tau and their interactions with time

^b^Adjusted for age, sex, APOE4, baseline Aβ a or Tau, vascular risk score, and their interactions with time

**Table 3:** Association of the baseline BMI strata with neuroimaging biomarker changes for 2 years

	**Estimate**	**95% CI**	***t* value**	***p* value**
**Dependent variable: Aβ retention**				
Baseline BMI strata x time	-0.004	-0.016 to 0.008	-0.653	.514
**Dependent variable: Tau deposition**				
Baseline BMI strata x time	-0.067	-0.118 to -0.017	-2.606	.011

*Abbreviations*: *Aβ* β-amyloid protein, *APOE* apolipoprotein e, *BMI* body mass index, *CI* confidence interval

Adjusted for age, sex, APOE e4, baseline Aβ or Tau and their interactions with time

**Table 4:** Results from sensitivity analyses for the association of the baseline BMI with neuroimaging biomarker changes for 2 years

	**Estimate**	**95% CI**	***t* value**	***p* value**
**Dependent variable: Aβ retention**				
Baseline BMI x time	0.0003	-0.003 to 0.002	-0.232	.817
**Dependent variable: Tau deposition**				
Baseline BMI x time	-0.016	-0.028 to -0.004	-2.551	.012

*Abbreviations*: *Aβ* β-amyloid protein, *APOE* apolipoprotein e, *BMI* body mass index, *CI* confidence interval

Adjusted for smoking status, alcohol intake status, and lifetime physical activity as well as age, sex, apolipoprotein e4, vascular risk score, baseline Aβ or tau and their interactions with time

### Association of BMI with cerebral Aβ and tau deposition change over two years stratified based on sex

A lower baseline BMI was associated with increased tau deposition over two years in men, but not in women (Table 5). As for Aβ changes, neither women nor men showed significant association between baseline BMI and cerebral Aβ changes over two years.

**Table 5:** Association of the baseline BMI with neuroimaging biomarker changes according to Sex

	**Female**			**Male**		
	**Estimate**	**95% CI**	***p* value**	**Estimate**	**95% CI**	***p* value**
**Dependent variable: global Aβ retention**						
Baseline BMI x time	0.001	-0.002 to 0.004	.671	-0.002	-0.005 to 0.001	.250
**Dependent variable: Global Tau deposition**						
Baseline BMI x time	-0.013	-0.029 to 0.003	.115	-0.027	-0.046 to -0.009	.007

*Abbreviations*: *Aβ* β-amyloid protein, *BMI* body mass index, *CI* confidence interval

Adjusted for age, APOE4, baseline Aβ or tau and their interactions with time

## Discussion

The present study found that a lower BMI was associated with greater increase of brain tau deposition over two years in cognitively healthy older adults. Further exploratory analyses showed that this association was significant in men, but not in women. In contrast, baseline BMI was not significantly associated with the change in cerebral Aβ deposition.

Our findings on the relationship between lower baseline BMI and greater increase in brain tau deposition are in agreement with previous reports of a cross-sectional association between lower BMI and higher CSF tau levels in older individuals [9, 13, 14, 40]. Although it is not easy to clearly explain the mechanisms underlying the relationship between lower BMI and greater increase in brain tau deposition, some possible explanations can be provided. First, the association between lower BMI and increased tau in the brain may be mediated by decreased leptin levels, a hormone synthesized from body fat that regulates appetite and energy metabolism [41]. Several laboratory studies have demonstrated that leptin reduces phosphorylated tau in in vivo and in vitro experiments [42–44]. This possibility of leptin mediation may further explain why the association is more prominent in males than females. As leptin expression is higher in subcutaneous than visceral fat [41, 45], it is more likely to be lower in thin males than thin females. Even at the same BMI, males have less subcutaneous fat than females [41, 46]. Second, alterations in insulin regulation may influence brain tau pathology [47]. Insulin inhibits tau hyperphosphorylation [48, 49], and plasma insulin can be transported via the blood–brain barrier into the cerebrospinal fluid [50]. Given people with low BMI have lower plasma insulin levels than those with higher BMI [51], decreased insulin levels in thin individuals may accelerate the brain deposition of pathological tau protein by ameliorating the insulin function to inhibit tau phosphorylation.

Additional exploratory analyses demonstrated male-specific association between lower baseline BMI and increased tau deposition over two years. The finding is generally in line with our previous report which showed a male-specific association between mid-life lower BMI and reduced AD-signature region cortical thickness [11]. Both findings may explain the neuropathological links underlying sex-specific association between BMI and AD dementia risk repeatedly shown by epidemiological studies [18, 19, 52].

We did not find a significant relationship between baseline BMI and longitudinal brain Aβ changes for all participants. This disagrees with previous cross-sectional findings for the association between lower BMI and higher Aβ deposition in cognitively healthy older individuals [9–12]. Given very gradual accumulation of Aβ in the brain [53], the two-year follow-up period may be relatively short to assess changes in Aβ deposition. Such short-term observations may affect the null finding for the association between BMI and changes in Aβ deposition.

Our finding for the relationship between lower late-life BMI and prospective increase in in vivo tau pathology is a novel one. Nevertheless, the present study had several potential limitations that should be addressed. First, as the proportion of participants with obesity (BMI over 30 mg/kg^2^) and underweight (BMI below 18.5 mg/kg^2^) was very small in our sample [3.1% (*n* = 6) and 1% (*n* = 2) of overall participants, respectively], it might be difficult to investigate the influence of higher BMI, obesity or very low BMI on the change in AD pathologies. Second, the first tau PET was performed at an average of 2.55 years (standard deviation 0.26 years) after BMI measurement at baseline, whereas the first amyloid PET was performed at baseline. This temporal gap may have influenced the results. However, when we controlled for the temporal gap as an additional covariate, the results did not change. Third, only a subset of participants (*n* = 45) underwent two tau PET scans, whereas all participants underwent two amyloid PET scans. Despite the smaller sample size for tau, we found a statistically significant relationship between BMI and change in tau deposition. This indicates that a small sample size may not be a critical issue. Nevertheless, a study with a larger sample size is required to confirm the sex-specific association between BMI and pathological changes in AD patients. Finally, mood status and various lifestyle factors may confound the association between BMI and changes in AD biomarkers. To minimize this possibility, we performed additional sensitivity analyses including smoking status, alcohol status, lifetime physical activity, and GDS as additional covariates and still obtained similar results. However, we could not control for food intake or dietary quality due to the lack of information.

## Conclusion

The present findings suggest that lower BMI in late life may predict or contribute to the progression of tau pathology over subsequent years in cognitively unimpaired older adults. Concerning the prevention of AD dementia or related cognitive decline, more attention needs to be paid to avoid being underweight in late life, particularly in men.

## Data Availability

The datasets generated and analyzed during the present study are not publicly available, owing to ethics considerations and privacy restrictions. Data might be obtained from the corresponding author after approval by the Institutional Review Board of the Seoul National University Hospital, South Korea.
